# Supplementary material for: γδT Cells Are Required for CD8+ T Cell Response to Vaccinia Viral Infection
Source: Front Immunol. 2021 Oct 8;12:727046. doi: 10.3389/fimmu.2021.727046 (PMC8531544; doi:10.3389/fimmu.2021.727046)
Supplement: Supplementary file 1 [file Presentation_1.pdf]

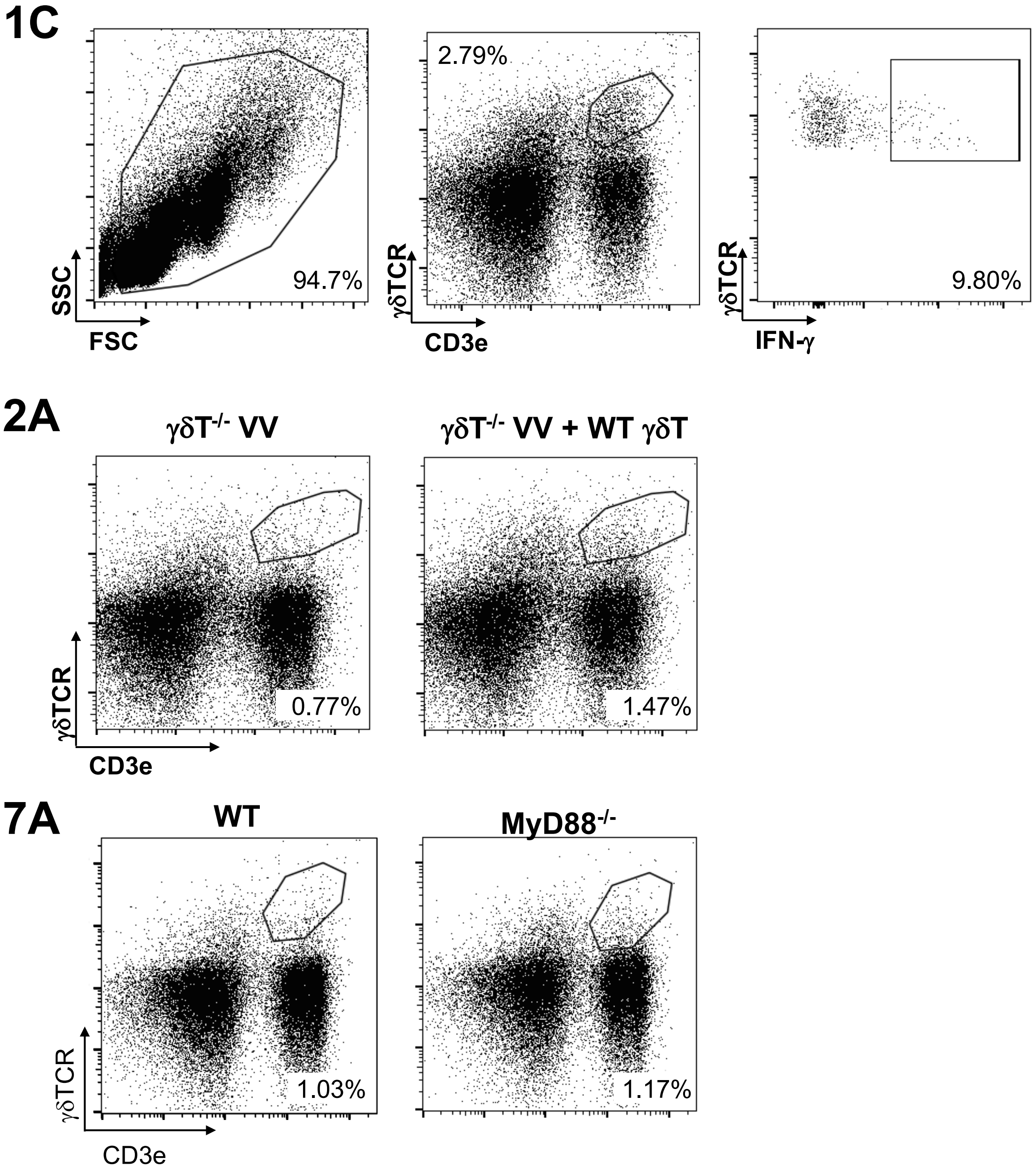

SUPPLEMENTAL FIGURE 1. Replicate dot plot representatives of figures 1C, 2A, and 7A, demonstrating individual cells of each population.
