# Supplementary material for: γδT Cells Are Required for CD8+ T Cell Response to Vaccinia Viral Infection
Source: Front Immunol. 2021 Oct 8;12:727046. doi: 10.3389/fimmu.2021.727046 (PMC8531544; doi:10.3389/fimmu.2021.727046)
Supplement: Supplementary file 2 [file Presentation_2.pdf]

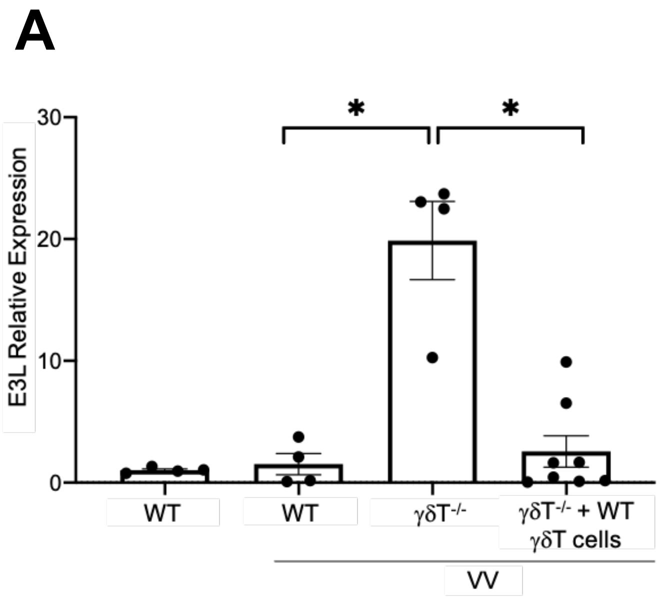

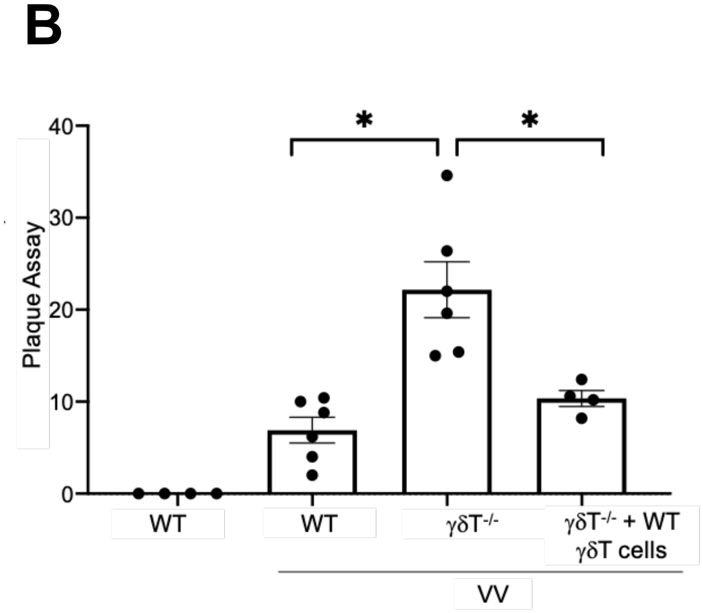

SUPPLEMENTAL FIGURE 2. WT and $\delta TCR^{-/-}$ mice were inoculated with $5x10^6$ pfu of VV intraperitoneally, with or without adoptive transfer of $1x10^6$ WT γδT cells. 3 days post-VV inoculation, the intraperitoneal space was washed with 1xPBS and assessed for viral load via **(A)** qRT-PCR for VV gene E3L and **(B)** plaque assay. Values are mean ± SEM, representative of 3 independent studies. ANOVA with post-hoc t-test, $^*P < 0.01$.
