# Supplementary material for: γδT Cells Are Required for CD8+ T Cell Response to Vaccinia Viral Infection
Source: Front Immunol. 2021 Oct 8;12:727046. doi: 10.3389/fimmu.2021.727046 (PMC8531544; doi:10.3389/fimmu.2021.727046)
Supplement: Supplementary file 3 [file Presentation_3.pdf]

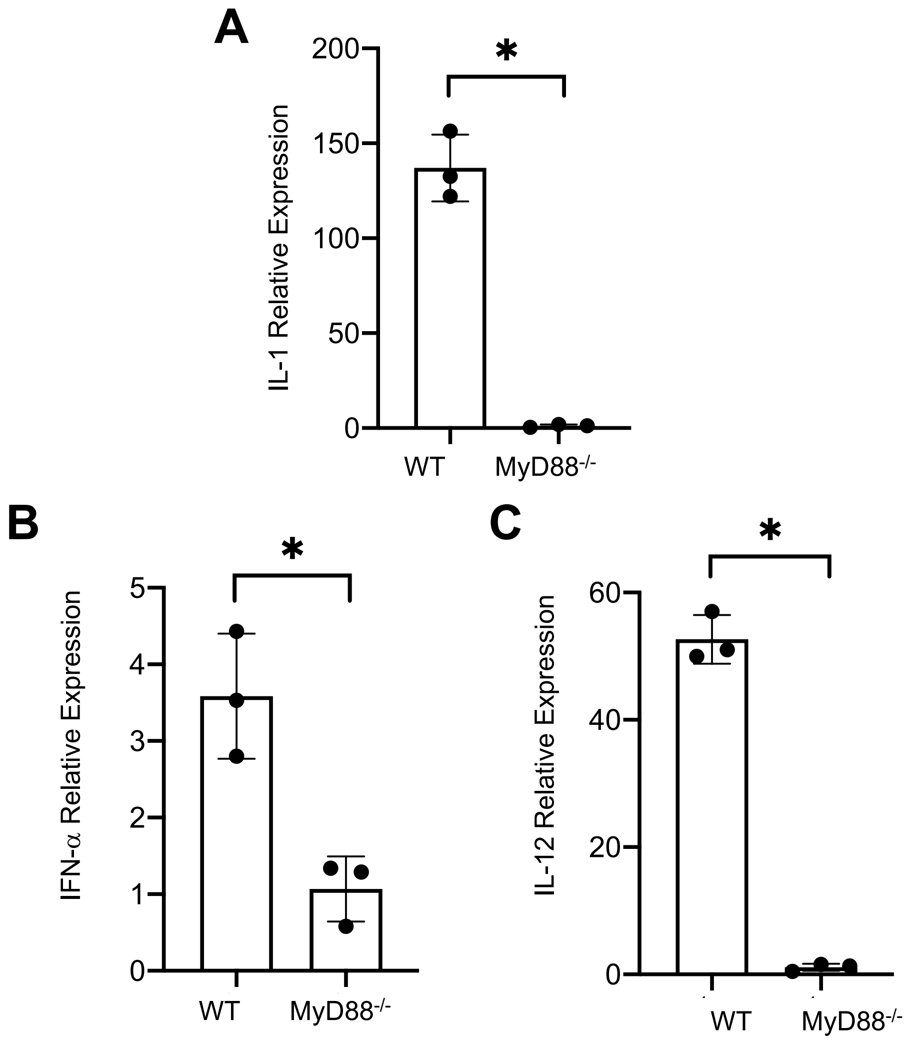

SUPPLEMENTAL FIGURE 3. Expression of signal 3 genes is different between WT and MyD88$^{-/-}$ mice inoculated with VV. Wild-type and *MyD88$^{-/-}$* mice were inoculated with VV intraperitoneally and 4 days post-inoculation, splenocytes were harvested. $\gamma\delta$T cells were isolated with MACS. Total RNA was extracted from the isolated cells and assessed by qRT-PCR for expression of **(A)** IL-1, **(B)** IFN-α, and IL-12a. Unpaired student T-test, *$^*P$ < 0.005. Each panel is representative of 3 independent studies.
