# Supplementary material for: γδT Cells Are Required for CD8+ T Cell Response to Vaccinia Viral Infection
Source: Front Immunol. 2021 Oct 8;12:727046. doi: 10.3389/fimmu.2021.727046 (PMC8531544; doi:10.3389/fimmu.2021.727046)
Supplement: Supplementary file 4 [file Presentation_4.pdf]

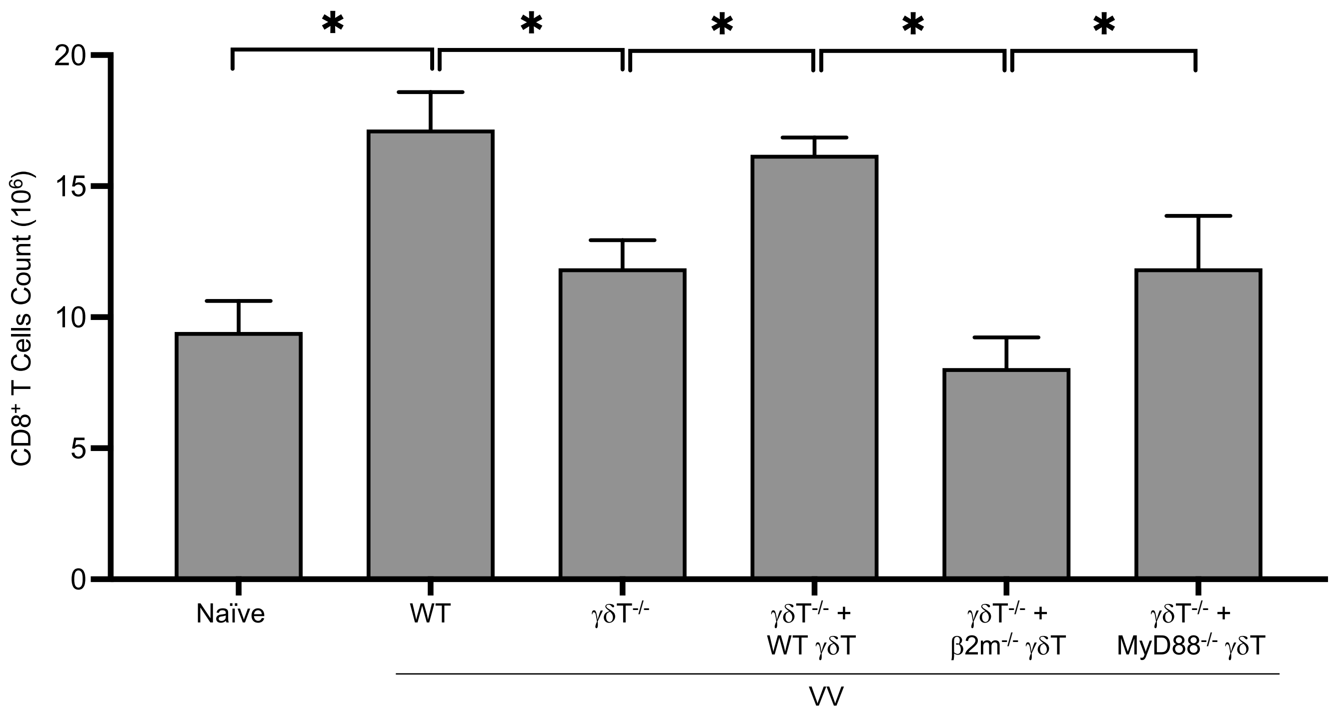

SUPPLEMENTAL FIGURE 4. WT and *δTCR*$^{-/-}$ mice were inoculated with $5x10^6$ pfu of VV intraperitoneally, with or without adoptive transfer of $1x10^6$ WT, *β2m*$^{-/-}$, or *MyD88*$^{-/-}$ γδT cells. 7 days post-VV inoculation, the spleen was obtained for absolute $CD8^+$ T cell count. Values are mean ± SEM, representative of 3 independent studies. ANOVA with post-hoc t-test, $*P < 0.05$.
